# Preschoolers' information search strategies: Inefficient but adaptive

**DOI:** 10.3389/fpsyg.2022.1080755

**Published:** 2023-01-04

**Authors:** Kai-Xuan Chai, Fei Xu, Nora Swaboda, Azzurra Ruggeri

**Affiliations:** ^1^MPRG iSearch, Max Planck Institute for Human Development, Berlin, Germany; ^2^Department of Psychology, University of California, Berkeley, Berkeley, CA, United States; ^3^Department of Cognitive Science, Central European University, Vienna, Austria; ^4^Department of Education, School of Social Sciences and Technology, Technical University of Munich, Munich, Germany

**Keywords:** information search, strategy, efficiency, active learning, cognitive development

## Abstract

Although children's sensitivity to others' informativeness emerges early in life, their active information search becomes robustly efficient only around age 10. Young children's difficulty in asking efficient questions has often been hypothesized to be linked to their developing verbal competence and growing vocabulary. In this paper, we offer for the first time a quantitative analysis of 4- to 6-year-old children's information search competence by using a non-verbal version of the 20-questions game, to gain a more comprehensive and fair picture of their active learning abilities. Our results show that, even in this version, preschoolers performed worse than simulated random agents, requiring more queries to reach the solution. However, crucially, preschoolers performed better than the simulated random agents when isolating the extra, unnecessary queries, which are made after only one hypothesis is left. When additionally isolating all the unnecessary queries, children's performance looked on par with that of the simulated optimal agents. Our study replicates and enriches previous research, showing an increase in efficiency across the preschool-aged years, but also a general lack of optimality that seems to be fundamentally driven by children's strong tendency to make unnecessary queries, rather than by their verbal immaturity. We discuss how children's non-optimal, conservative information-search strategies may be adaptive, after all.

## Introduction

Asking questions is one of the most powerful tools for children to actively learn about and engage with the world around them (see Callanan and Oakes, 1992; Chouinard, 2007; Ruggeri et al., 2021), allowing children to formulate complex queries that target the specific pieces of information desired (e.g., Hickling and Wellman, 2001; Greif et al., 2006; Frazier et al., 2009), to explore the immaterial world of concepts, emotions and social relationships (e.g., Wellman, 2011; Harris, 2012), and to target multiple features of objects/events—present or absent alike—at once (Mosher and Hornsby, 1966; Herwig, 1982; Ruggeri and Feufel, 2015; Ruggeri and Lombrozo, 2015; Ruggeri et al., 2016). Such developing competence in searching for information has been traditionally investigated using the 20-questions game (e.g., Mosher and Hornsby, 1966; for a review, see Jones et al., 2020), in which children are tasked to identify the correct answer (e.g., “Why was the man late for work today?”) within a given *hypothesis space* (*HS*) by asking as few yes–no questions as possible (e.g., “Was the man late because he woke up late?”; Ruggeri et al., 2017)^1^.

However, among all the information-gathering tools available to children—including pointing, joint attention and physical exploration, asking questions is the one that emerges the latest, steadily increasing in sophistication from early childhood to adulthood. On the one hand, young children clearly have difficulties in asking efficient questions, and this has often been hypothesized to be linked to their developing verbal competence and growing vocabulary (see Herwig, 1982; Ruggeri and Feufel, 2015; Ronfard et al., 2018; Ruggeri et al., 2021; Swaboda et al., 2022). On the other hand, albeit being a powerful information-seeking tool, some work on language development find that asking questions does not necessarily relate to any other aspect of learning (for a review, see Callanan et al., 2020). This may have to do with the consideration that questions (and in particular children-directed questions) often do not have clear teaching intentions and learning effects, but are rather meant to be engaging (e.g., “Where's your nose”; see work by Golinkoff, 1986). In other words, children's information search competence could be underestimated if they are requested to rely on generating verbal questions. In this paper, we examine 4- to 6-year-olds' information search competence by using a non-verbal version of the 20-questions game, to gain a more clear, comprehensive and fair picture of their active learning abilities (see also Ruggeri, 2022).

### The developmental trajectory in information search competence

Children begin to demonstrate meaningful information search patterns very early in life (for a review, see De Simone and Ruggeri, 2022). Infants tend to direct their attention to events with intermediate complexity (supposedly to avoid wasting effort in processing overly simple or complex events; Kidd et al., 2012), prefer to interact with knowledgeable social partners (see Csibra and Gergely, 2009; Bazhydai et al., 2020), and are more likely to explore objects/events that violate their expectations and naïve theories (e.g., Stahl and Feigenson, 2015; Dunn and Bremner, 2017; Sim and Xu, 2017a). Preschoolers selectively engage and spend more time exploring what they are uncertain about or what does not fit their prior beliefs (e.g., Schulz and Bonawitz, 2007; Bonawitz et al., 2012; Legare, 2012), preferentially rely on and query the most reliable informants (for reviews, see Mills, 2013; Harris et al., 2018), and start to use the knowledge of informative intervention to disambiguate causal structures (Schulz et al., 2007; Cook et al., 2011; McCormack et al., 2016; see also Sim and Xu, 2017b; Lapidow and Walker, 2020; Sobel et al., 2022). These findings suggest that children's non-verbal information search is already pretty efficient and rather sophisticated at a very young age.

However, other findings trace a much more protracted developmental trajectory, with children showing mature, adult-like active learning patterns only by late childhood. For example, Ruggeri et al. (2016) offer a more fine-grained computational analysis of children's developing question-asking competence, indicating that children do not begin to systematically generate the most effective questions until age 7 (see also Mosher and Hornsby, 1966; Herwig, 1982; Ruggeri et al., 2021), and they do not demonstrate robust adult-like inquiry patterns until around age 10 (see also Ruggeri and Feufel, 2015).

### Reasons for inefficiency

Previous work has hypothesized and tested several factors potentially responsible for the observed developmental change (see Jones et al., 2020 for a review). First, young children's difficulty in asking efficient questions may be linked to their ability to recognize and exploit the abstract, hierarchical structure of the hypothesis space (Jones et al., 2021). Indeed, the ability to identify and flexibly categorize objects based on alternative features predicts how well 4- to 6-year-olds generate effective questions (Legare et al., 2013), and scaffolding higher-level representations facilitates children's ability to ask efficient questions (Ruggeri and Feufel, 2015).

Second, the developmental differences in search efficiency may also be due to children's growing ability to correctly update the feedback received (Kachergis et al., 2017), to focus on the most relevant cues (see Davidson, 1991a,b; Betsch et al., 2016, 2018), or to implement efficient rules to decide when to stop searching for more information (Mata et al., 2011; Ruggeri et al., 2016). For example, Ruggeri et al. (2016) demonstrated that children from ages 7 to 10 are considerably more likely than adults to keep making queries beyond the point at which a single hypothesis remains—i.e., the solution could be reached. Along these lines, Kachergis et al. (2017) showed that 8- to 10-year-olds often mistakenly update the hypothesis space, which was based on the feedback of feature-based queries that target multiple hypotheses.

Third, the developmental change may be simply accounted for by children's improving verbal skills, which underlie their ability to *formulate* informative questions. For instance, when verbal requirements are reduced or scaffolded, even 4-year-olds can generate questions targeting higher-order categories, enabling them to target multiple alternatives simultaneously (Ruggeri et al., 2021). Indeed, when they do not have to generate questions from scratch, the performance of primary school children strongly improves (Herwig, 1982; Ruggeri and Feufel, 2015), and even 5-year-olds can identify the more informative question between two given questions (Ruggeri et al., 2017). A similar effect has been demonstrated in research on children's scientific reasoning, showing that selecting an intervention to test the effect of a certain variable is easier for preschoolers than coming up with an intervention from scratch (e.g., van der Graaf et al., 2015; Lapidow and Walker, 2020; Moeller et al., 2022; see also Bramley et al., 2022).

### The present study

In this study, we offer for the first time a quantitative analysis of 4- to 6-year-olds' information search competence. To examine preschoolers' information search efficiency when the verbal challenges posed by the tasks used in previous research are stripped away, we implemented a non-verbal version of the 20-questions game (see Ruggeri et al., 2016, Exp.2) in which children were not required to *articulate* questions, even a more generally yes–no questions, but could select or indicate individual objects (potentially by pointing) they wanted to ask about^2^. We also reduced the task demands by scaffolding children's object categorization with a card-sorting familiarization task and providing the memory aid throughout the searching (see Section Design and procedure).

Learning about the world by asking questions might be the best approach if the questioner has a good intuition of the to be learned in the first place. But for preschoolers, whose learning environment is usually serendipitous, they probably may not own organized prior knowledge for the learning task. Indeed, previous work indicates that preschoolers almost exclusively ask questions targeting individual objects, rather than *categories* of objects (e.g., Mosher and Hornsby, 1966; Herwig, 1982). Such bottom-up, exploratory search strategy could be more practical and adaptive for young children, as it can provide evidence overriding existing questionable knowledge and give rise to entirely new concepts and conceptual structures (see Piaget, 1954; Carey, 1985; Gopnik and Wellman, 2012; Xu, 2019). We thus claim that our object-selection paradigm may be more ecologically valid and in line with preschoolers' spontaneous search approach, compared to the previously used 20-questions game, and does resemble those used in research examining the development of scientific inquiry skills (see Bramley et al., 2022).

Based on the above-reviewed prior work, which indicates that children start to demonstrate efficient information-search patterns once they are scaffolded, we expected a developmental improvement in the efficiency of preschoolers' queries in the less-demanding task designed in the current study, reflected in their *number of queries* required to narrow down the hypothesis space and reach the solution. Crucially, we conducted a thoroughly exploratory analysis to shed light on the factors underlying the hypothesized developmental differences. In particular, we examined children's tendency to make uninformative queries during their search. Finally, to better evaluate children's performance, we compared it to that of simulated normative models, which offer the best and chance-level performance benchmarks.

## Methods

### Participants

Participants were 67 children aged 4–6 (31 females, 36 males; *M* = 67.2 months; *SD* = 11.16 months; range: 48–88 months), recruited by random approach at local museums in San Francisco and Berkeley, California (sample size determined based on Ruggeri et al., 2016 and convenience). The parents were not asked to disclose their socioeconomic status, and the racial information of the children was not recorded, either. However, the visitors of these partner museums are racially diverse and from representative social classes. The experiment was conducted in a quiet room of the museum and was video-recorded. Eleven additional children withdrew before the end of the experimental session (*n* = 10) or due to technical difficulties (*n* = 1). Written informed consent of legal guardians was obtained before participation. Children were asked for verbal consent and received stickers for their participation. The study was approved by the IRB of the University of California, Berkeley (protocol: CPHS#:2010-01-631) and was conducted in accordance with the ethical principles of *The Belmont Report*.

### Design and procedure

**Figure 1:**
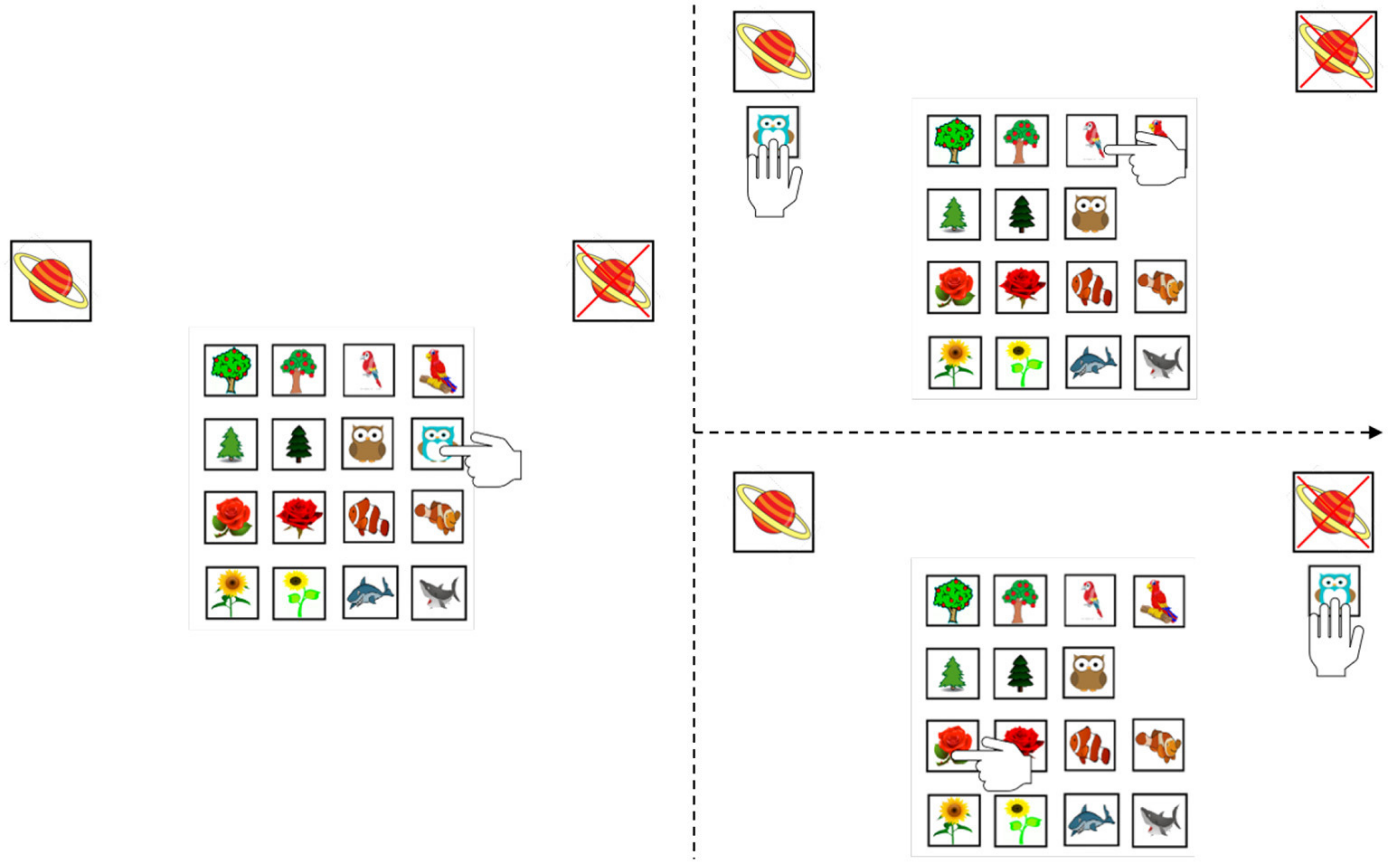
Stimuli display and illustration of the search path. These 16 cards are grouped into three hierarchical levels in a 4-by-4 grid display: 2 superordinate levels (i.e., animals and plants), 4 basic levels (i.e., fish, birds, trees and flowers), and 8 subordinate levels (i.e., owls, parrots, clownfish, shark, pine/Christmas trees, apple trees, roses and sunflowers).

Children were presented with 16 cards depicting animals and plants from the “Planet Apres” scenario developed by Ruggeri et al. (2016). These objects were designed such that they could be grouped into nested hierarchical categories (see Figure 1). To highlight such structure, the experimenter laid out the 16 cards in front of the children, in random order, and said “These cards can be grouped into two big groups.” The experimenter then sorted the cards into the two superordinate categories (i.e., animals and plants) and prompted the children to label them (“What are these? These are all...?”). The same procedure was repeated (“We can have some more groups. The groups of the [xxx] can be split into two groups.”)—first sorting both superordinate-level groups into the four basic-level groups, and then sorting the four basic-level groups into the eight subordinate-level groups. After each sorting step, the experimenter rearranged the cards to reflect the hierarchical organization of the different groups, eventually producing the 4-by-4 grid display used in the test (see Figure 1).

After the sorting and reorganizing was completed, children were told that some of the objects depicted on the cards could be found on a far-away planet called “Apres,” and that their task was to find out which *kind of objects* could be found there by inquiring about the objects one by one. The experimenter prompted the children to select objects by saying “What is the first/next object you want to know about? Do you want to point at it?” The experimenter then provided yes or no feedback to each query, moving the inquired card to the “yes” or “no” piles, indicated by two cards depicting Planet Apres or a crossed-out Planet Apres, respectively (see Figure 1). After each query, the experimenter drew the children's attention to the two piles, pointing out that “we know that this/these object(s) is/are on planet Apres (*pointing to the yes-side*), and we know that this/these object(s) is/are *not* (*pointing to the no-side*). Do you think you know which kind of objects could be found on Planet Apres?” If children responded yes, they were prompted to provide a category label (e.g., “The owls!”) or to point at all the objects that could be the answer (i.e., all the owls). Because we told children that the solution includes more than one of the given objects, they knew the solution could not be, e.g., “only the blue owl.” If children said they did not know or they provided an incorrect answer, they were encouraged to inquire further (“Mmm...why don't you select another object to ask?”). This procedure was repeated until the children provided the correct solution. If children had already sorted all cards without having yet provided the correct solution, the experimenter pointed to the group of objects under the yes-pile and asked “Why do you think these are all on Planet Apres? Because they are all...” The correct solution was pseudo-randomized across participants.

To motivate children to minimize the number of queries and to be as efficient as possible, they were given 16 tokens at the beginning of the experiment and had to pay one token for every object they wanted to query. The tokens children had left over at the end of the game could be exchanged for stickers. Those children who had used up all their tokens by the end of the session still received one thank-you sticker.

### Simulations

We simulated a total of 10,000 optimal agents and 10,000 random agents, using R language (also for the data analysis), to sketch the best and the chance performance of our task respectively. An *optimal* agent queries, at each step of the search, the object that can maximally partition the hypothesis space. For example, suppose that an agent started by selecting the blue owl. If the feedback was “yes,” the solution could be “only owls,” “all birds,” or “all animals” are on planet Apres. In this case, the most informative follow-up selection would target one of the remaining animals; selecting the brown owl or one of the plants would not be helpful regarding the reduce of the *HS*. If the feedback was “no,” the solution could not be “owls,” “birds,” or “animals.” In this case, the most informative follow-up selection would target one of the plants; selecting the other owl would be uninformative; selecting one of the other animals would be less informative because it eliminates fewer hypotheses. A *random* agent queries a random object at each step of the search, regardless of how much the *HS* it reduces. Crucially, in our simulations both the optimal and the random agent stop searching when the *HS* has been narrowed down to one hypothesis, that is, when the solution is found.

## Results

### Efficiency of preschoolers' information search

**Figure 2:**
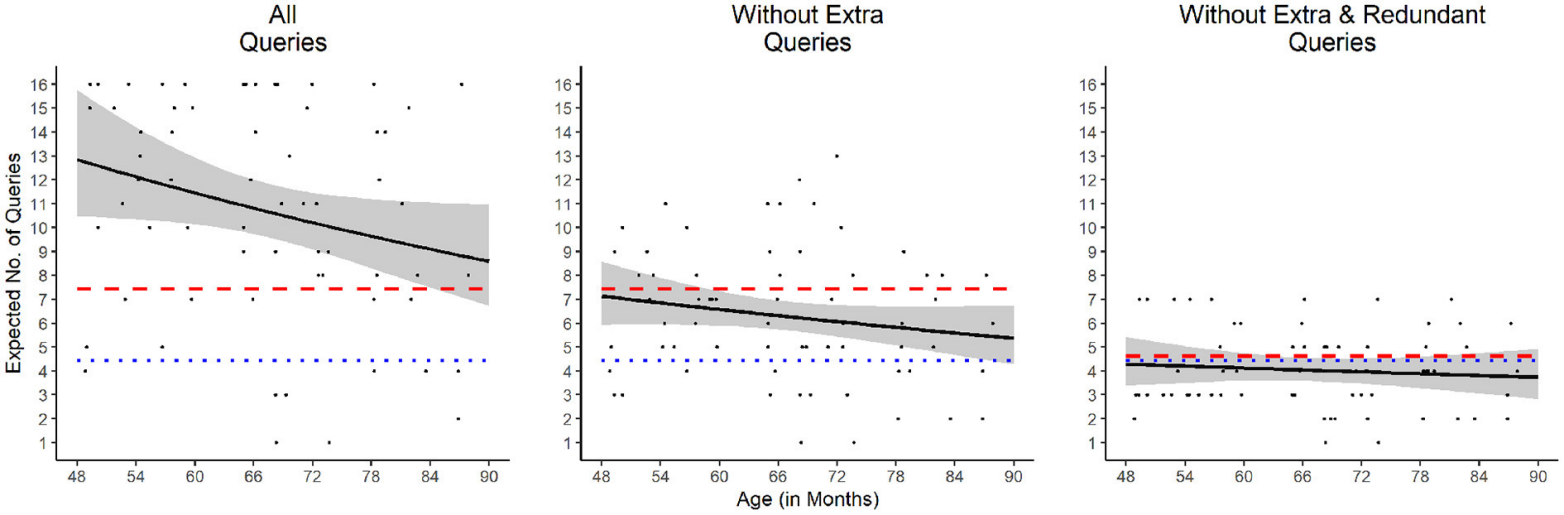
Expected number of queries as a function of age. The shaded areas represent 95% confidence intervals. The blue dotted lines represent the performance of the simulated optimal agents, and the red dashed lines represent the performance of the simulated random agents. Note that the performance of the simulated optimal and random agents are always significantly different from each other. Only the left figure was fitted by a Negative Binomial model, while the other figures were fitted by Poisson models.

Overall, preschoolers made an average of 10.75 queries before reaching the solution. This is significantly more than what was required, on average, by both the simulated optimal and random agents, *p*s < 0.001 (Bonferroni-corrected; see Table 1). To examine the developmental trajectory, we fitted a negative binomial (*NB*) model, due to the characteristic of the over-dispersed count data, with number of queries as the response variable and age (in months) as the predictor. The model showed that age accounted for a significant amount of variance in the number of preschoolers' queries [likelihood ratio [LR] $\chi_{\left(1\right)}^{2}$ = 3.871, *p* = 0.049, McFadden's pseudo-*R*^2^ = 0.010]. Indeed, age showed a significant predictive effect, *b* = –0.0096, 95% CI [–0.01914, –0.00004], *p* = 0.047, with the expected number of preschoolers' queries decreasing by 10.85% for 1 year of growth (see Figure 2, left).

**Table 1:** Mean scores and standard errors of number of queries by agents (participants and simulated random and optimal agents).

**Agents**	***n***	**All queries**	**Without extra queries**	**Without extra and redundant queries**
Preschoolers	67	10.75 (0.549)	6.28 (0.345)	4.03 (0.203)
Simulation (random)	10,000	7.44 (0.031)	7.44 (0.031)	4.61 (0.017)
Simulation (optimal)	10,000	4.44 (0.015)	4.44 (0.015)	4.44 (0.015)

### Unnecessary queries

Our analyzes above seem to suggest that preschoolers' performance is not only suboptimal but even worse than what could be expected from an agent selecting objects at random. In what follows, we deconstruct children's performance, isolating and examining the potential reasons underlying the lack of efficiency observed in their search.

Almost 62.5% of preschoolers' queries did not narrow down the hypothesis space and were therefore *unnecessary* from an optimal-search perspective. Indeed, only 7.46% of the preschoolers in our study did not make any unnecessary queries. Overall, preschoolers made an average of 6.72 unnecessary queries (*SE* = 0.516) before reaching the solution. An *NB* model was used and showed that age did not account for a significant amount of the variance (*p* = 0.092).

Children's unnecessary queries may be related to their strong tendency to continue making queries even when there was only one viable hypothesis left—a failure in their *stopping rules* (see also Ruggeri et al., 2016)—or from a more general inefficiency in asking informative questions to narrow down the *HS*. We distinguish between unnecessary queries performed *after* or *before* the *HS* had been narrowed down to one hypothesis, which we refer to, respectively, as *extra* queries (i.e., queries that are uninformative because there are no alternatives left), and *redundant* queries, which include queries targeting objects whose feedback could be inferred from previous queries (e.g., targeting the second owl, when we already query the first owl; targeting a flower when we already know that one shark is on planet Apres).

#### Extra queries

Overall, 77.61% of the preschoolers made at least one extra query after only one viable hypothesis was left. In particular, they made an average of 4.46 extra queries (*SE* = 0.449), which is significantly more than that of the 7-year-olds (*M* = 2.23) from previous studies implementing a very similar paradigm (Ruggeri et al., 2016, Exp.2) [*t*_(66)_ = 4.97, *p* < 0.001, Cohen's *d* = 0.608]. An *NB* model showed that age did not account for a significant amount of variance in the number of extra queries (*p* = 0.217).

Crucially, if we consider only the queries made up to the point at which the *HS* had been narrowed down to one hypothesis, that is, stopping searching when the solution is reached—which both simulated models were designed to do—preschoolers required an average of 6.28 queries to reach the solution. Although still significantly more than what was required by the simulated optimal agents, this number of queries is significantly less than what was required by the simulated random agents, *p*s < 0.001 (Bonferroni-corrected; see Table 1). A Poisson model was used and revealed that there is no significant effect of age (*p* = 0.125; see Figure 2, middle).

#### Redundant queries

Overall, 73.13% of the preschoolers made at least one redundant query before they provided the correct solution. In particular, preschoolers made an average of 2.25 redundant queries (*SE* = 0.275), which is less than those made by our random agents [*M* = 2.83, *SE* = 0.025; *t*_(67.121)_ = 2.097, *p* = 0.040, Cohen's *d* = 0.257]. An *NB* model was used and showed that there is no effect of age on the number of redundant queries (*p* = 0.227).

We further analyzed the data by isolating all the unnecessary queries (both the extra and the redundant queries) from both children's and the simulated random agents' performance. After this isolation, preschoolers required an average of 4.03 queries to reach the solution—fewer than what was required by the new simulated random agents, *p* = 0.007, but not significantly different from what was required by the simulated optimal agents, *p* = 0.095 (see Table 1)^3^. A Poisson model was used and indicated that age did not account for a significant amount of the variance (*p* = 0.547; Figure 2, right).

#### Feedback on unnecessary queries

Although, from an optimal-search perspective, unnecessary queries are uninformative as they make little contribution to narrowing down the hypothesis space, preschoolers might still make these queries in the hope of receiving positive, confirmatory feedback, as the experience of confirming an identified hypothesis could be rewarding in itself (see Nickerson, 1998). Overall, the large majority of preschoolers' unnecessary queries (72.24% of the extra queries and 87.42% of the redundant queries) received negative/no feedback instead of positive/yes feedback; most of their unnecessary queries (65.56% of the extra queries and 53.64% of the redundant queries) targeted the twin object of what has been queried (e.g., targeting the second owl while the first owl has already been queried) instead of the object out of the hypothesis space (e.g., targeting a flower when one shark is already confirmed to be on planet Apres).

Crucially, a logistic regression was used to analyze preschoolers' likelihood of making an unnecessary query again predicted by the feedback received (positive/yes feedback vs. negative/no feedback) and the object queried (the twin object of what has been queried vs. the object out of the hypothesis space), showing that the feedback accounted for a significant amount of the variance [LR $\chi_{\left(1\right)}^{2}$ = 3.866, *p* = 0.049, McFadden's pseudo-*R*^2^ = 0.008]. In particular, we found that receiving positive feedback marginally significantly increased children's likelihood of making unnecessary queries, compared to receiving negative feedback, *OR* = 1.697, *p* = 0.056.

## Discussion

The present study explored the efficiency of children's information-search strategies. In particular, we examined preschoolers' search efficiency using a non-verbal version of the traditional 20-questions game, which does not require children to generate questions from scratch. By ruling out one of the potential confounding effects, i.e., preschoolers' verbal abilities, we aimed to paint a fairer picture of the efficiency of preschoolers' information-search strategies.

We found a general developmental improvement in preschoolers' performance, as reflected in the number of queries they required to solve the game. This finding conceptually replicates the results from Ruggeri et al. (2021), which showed that with scaffolding older preschoolers ask fewer questions than younger preschoolers to solve a traditional (i.e., verbal) 20-questions game. Our work also supplements previous work (Mosher and Hornsby, 1966; Ruggeri and Feufel, 2015; Ruggeri et al., 2016), suggesting that the developmental improvement in information-search efficiency can be observed from around age 4. Nevertheless, we found that preschoolers' performance was generally very poor—even lower than that achieved by a model selecting objects at random.

The pervasive inefficiency in children's information search observed in our study is somehow surprising in light of previous research showing that children can implement efficient active learning strategies already from age 2 (Ruggeri et al., 2019). In particular, children's tendency to ask unnecessary queries is in contrast with previous results indicating that young children tend to be rather *overconfident* in what they have learned (e.g., Finn and Metcalfe, 2014; Salles et al., 2016). However, our results are in line with research on children's decision making, showing that younger children tend to be more exhaustive in their predecisional search than older children (Davidson, 1991a,b).

Our results demonstrate that preschoolers' inefficiency is not exclusively due to the verbal and categorization competence, as well as memory capacity, required by the paradigms implemented in previous work. Instead, our findings suggest that their inefficiency may be mainly due to their tendency to perform unnecessary queries (see also Ruggeri et al., 2016). In particular, when isolating the extra queries (e.g., those made after the hypothesis space had already been narrowed down to one hypothesis, which our simulated agents were designed to do), we found that preschoolers did indeed require fewer queries than the random agents, though still more queries than the optimal agents. When additionally isolating children's redundant queries, that is, considering only informative queries that reduce the hypothesis space, preschoolers' performance looked on par with that of the simulated optimal agents. Intriguingly, our results to some extent also support the hypothesis that the developmental trajectory in children's search efficiency is mostly driven by their decreasing tendency to perform unnecessary queries (Ruggeri et al., 2016). Indeed, the developmental trend flattens considerably when isolating the extra queries, and becomes completely flat when isolating all unnecessary queries.

Why did the preschoolers tend to perform unnecessary queries? It could be explained by children's developing uncertainty-monitoring abilities (see Lyons and Ghetti, 2011; Ghetti et al., 2013), which might make it difficult for them to monitor and correctly update the hypothesis space (see Kachergis et al., 2017), and to stop searching when the hypothesis space had been successfully narrowed down (see Ruggeri et al., 2016). Indeed, the 4- to 6-year-olds in our sample made more extra queries than the 7-year-olds from Ruggeri et al. (2016, Exp.2). However, note that they did not make redundant queries as arbitrarily as our simulated random agents, revealing that already by age 4 children are to some extent sensitive to changes in the hypothesis space. Another interpretation is that the rewarding experience of confirming an identified hypothesis reinforces children to make unnecessary queries (see Nickerson, 1998). Other work suggests a similar pattern in children's causal learning, showing that 5- to 7-year-olds often select confirmatory interventions, i.e., actions that are expected to produce an effect in line with their initial hypotheses (e.g., McCormack et al., 2016; Meng et al., 2018)—an inefficient but potentially adaptive *positive testing strategy* (Klayman and Ha, 1987; Coenen et al., 2015). This interpretation is partially supported by our exploratory analysis, showing that receiving positive feedback significantly increased children's likelihood of making unnecessary queries again. Lastly, it is worth noting that the developmental change observed could not be exclusively driven by memory improvements, as the modified paradigm we used rather minimized task demands. Thus, it is still an open question what is underlying children's developmental improvements in active search?

Crucially, our results also lead us to speculate that young children may employ different strategies to solve the problem than we expected, suggesting that they may favor more *conservative* strategies that search for information and explore intensively and rather frugally. If, from a more computational benchmark, such a strategy is clearly inefficient, there are many reasons why being prudent is highly desirable.

First, looking for confirming evidence could indeed make very much sense when there is uncertainty about the hypothesis space, the feedback one has received, or the robustness of what is being learned. Indeed, when children subjectively perceive the decision space is highly dispersed, they prefer to apply a compensatory, exhaustive information-search strategy—integrating all available information (see Betsch et al., 2016). Moreover, Legare et al. (2013) found that the preschoolers (4- to 6-year-olds) who asked more confirmatory questions performed better overall in a 20-questions game, suggesting that the confirmatory strategy, although inefficient, may be useful. Future work could use the current non-verbal paradigm with smaller search space to examine whether young children would be more efficient when the uncertainty perceived is not relatively high for them. Also, with appropriate simplification, future work could conduct multiple rounds of the task that manipulates the individual objects of the same categories, the categories with the same hierarchy, or the target category (i.e., the correct solution) within the same stimuli, to further track the effect of stimuli novelty/familiarity and the course of short-term learning.

Second, it is still an open question whether children understand the task the same way adults do. Although we incentivized preschoolers to solve the task in as few queries as possible, it is likely that preschoolers were motivated by goals other than efficiency maximization. For example, they could have been more interested in socially engaging with the experimenter (see also Jaswal and Kondrad, 2016) or in enhancing the chances for long-term retention of the stimuli (Stanciu et al., under review)^4^, or interested in the experience altogether. Future work could employ a non-social paradigm (e.g., a self-administered tablet game) or a task with less attractive stimuli to rule out these possible confounding factors.

It is important to note that some of the effects we found were fairly weak or only marginally significant. It is possible that our sample size was not large enough to detect appropriate effect sizes, given that it was initially determined based on some expected main effects rather than on the more exploratory analyzes we then conducted. In this sense, future work should try to reproduce and extend these findings.

## Conclusion

To conclude, the present study replicated and extended previous work on children's information search using a non-verbal version of the 20-questions game. We show an increase in efficiency across the preschool years, but also a general lack of optimality, which seems to be fundamentally driven by children's strong tendency to make unnecessary queries. Overall, as novice learners in an uncertain, noisy world, being prudent and preferring accuracy at the expense of efficiency, can be quite adaptive—after all, why should a 4-year-old care about being time- or resource-efficient?

## Data availability statement

The raw data supporting the conclusions of this article will be made available by the authors, without undue reservation.

## Ethics statement

The studies involving human participants were reviewed and approved by the University of California, Berkeley. Written informed consent to participate in this study was provided by the participants' legal guardian/next of kin.

## Author contributions

AR and FX contributed to the conception and design of the study. NS and AR organized the database. K-XC and NS performed the statistical analysis. K-XC and AR wrote the first draft of the manuscript. NS wrote sections of the manuscript. All authors contributed to the manuscript revision, read, and approved the submitted version.

## Footnotes

^1^Although this game may appear to be constrained at first glance, especially if compared to more naturalistic open-ended dialogues, it is a classic example of a sequential, binary information search problem that one encounters fairly often in everyday life and throughout the lifespan. For this reason, real-world decision-making, categorization, and causal inference tasks have often been modeled with fast and frugal trees that involve this sequential, binary branching (e.g., Berretty et al., 1997, 1999; Martignon et al., 2008). In this sense, studying children's performance on the 20-questions game is a good compromise between experimental tractability and real-world generalizability (see Jones et al., 2020).

^2^We claim that this paradigm is non verbal because “asking” and “formulating” questions from scratch is not necessary in this paradigm. In this sense, children's performance in this task is independent on their *expressive* language abilities, although their *receptive* language abilities are inevitably involved.

^3^Preschoolers' performance, considering only the informative queries, is numerically better than simulated optimal agents' because some preschoolers (10 out of 67) guessed the solution without narrowing down the *HS* to one hypothesis.

^4^Stanciu, O., Jones, A., Metzner, N., Fandakova, Y., Ghetti, S., and Ruggeri, A. (under review). Inefficient learners can be good learners: The (in)efficiency of children's search strategies selectively supports memory.
